# Longitudinal Analysis of Mitochondrial Function in a Choline-Deficient L-Amino Acid-Defined High-Fat Diet-Induced Metabolic Dysfunction-Associated Steatohepatitis Mouse Model

**DOI:** 10.3390/ijms25116193

**Published:** 2024-06-04

**Authors:** Akiko Yamada, Akira Watanabe, Atsushi Nara, Naozumi Ishimaru, Kosuke Maeda, Yusuke Ido, Kazumasa Kotake, Masatake Asano, Yasuo Shinohara, Takenori Yamamoto

**Affiliations:** 1 Department of Pathology, Nihon University School of Dentistry, Chiyoda-ku, Tokyo 101-8310, Japan; 2 Institute for Genome Research, Tokushima University, Kuramoto, Tokushima 770-8503, Japan; 3 Faculty of Pharmaceutical Sciences, Tokushima University, Shomachi, Tokushima 770-8505, Japan; 4 Department of Oral Pathology, Graduate School of Medical and Dental Sciences, Tokyo Medical and Dental University, Bunkyo-ku, Tokyo 113-8549, Japan; 5 Division of Molecular Target and Gene Therapy Products, National Institute of Health Sciences, Kawasaki-ku, Kanagawa 210-9501, Japan

**Keywords:** metabolic dysfunction-associated steatohepatitis, mitochondria, FoF_1_–ATPase, permeability transition, liver fibrosis

## Abstract

Metabolic dysfunction-associated fatty liver disease (MAFLD) is one of the most common chronic liver diseases worldwide. Some patients with MAFLD develop metabolic dysfunction-associated steatohepatitis (MASH), which can lead to severe liver fibrosis. However, the molecular mechanisms underlying this progression remain unknown, and no effective treatment for MASH has been developed so far. In this study, we performed a longitudinal detailed analysis of mitochondria in the livers of choline-deficient, methionine-defined, high-fat-diet (CDAHFD)-fed mice, which exhibited a MASH-like pathology. We found that FoF_1_–ATPase activity began to decrease in the mitochondria of CDAHFD-fed mice prior to alterations in the activity of mitochondrial respiratory chain complex, almost at the time of onset of liver fibrosis. In addition, the decrease in FoF_1_–ATPase activity coincided with the accelerated opening of the mitochondrial permeability transition pore (PTP), for which FoF_1_–ATPase might be a major component or regulator. As fibrosis progressed, mitochondrial permeability transition (PT) induced in CDAHFD-fed mice became less sensitive to cyclosporine A, a specific PT inhibitor. These results suggest that episodes of fibrosis might be related to the disruption of mitochondrial function via PTP opening, which is triggered by functional changes in FoF_1_–ATPase. These novel findings could help elucidate the pathogenesis of MASH and lead to the development of new therapeutic strategies.

## 1. Introduction

Metabolic dysfunction-associated fatty liver disease (MAFLD) is a widely recognized liver disorder associated with metabolic syndrome, and its prevalence is increasing globally. MAFLD affects approximately 30% of the population in Western countries [1]. This disease can be classified into fatty liver disease, which does not progress to severe disease despite the presence of fatty liver, and metabolic dysfunction-associated steatohepatitis (MASH), which progresses to severe and irreversible diseases, such as hepatocellular carcinoma (HCC), via steatohepatitis and liver fibrosis. In fact, approximately 40% of patients with MASH eventually develop HCC [2]. Currently, there is no effective treatment for MASH, and lifestyle modification via exercise and diet is the primary treatment strategy because the mechanisms underlying the development of MASH are not well understood. Therefore, elucidating the molecular mechanisms of MASH pathogenesis is critical for its treatment and prevention.

Previous studies have suggested that the pathological progression of MASH is closely related to the development of liver fibrosis, an irreversible condition caused by inflammation due to various factors, such as lipids, enteric endotoxins [3], oxidative stress [4], and inflammatory cytokines [5]; this is known as the multiple parallel hit hypothesis [6]. However, the molecular mechanisms leading to liver fibrosis are poorly understood. Appropriate experimental models can be used for elucidating these molecular mechanisms, but animal models that reflect the pathogenesis of MASH in humans have not yet been established, making research in this field difficult. Animal models of fatty liver and hepatitis, representing the early stages of MASH, have been extensively studied [7]. Several animal models of MAFLD/MASH exhibit impaired mitochondrial function in the liver; thus, understanding the relationship between hepatic mitochondrial dysfunction and the development of MAFLD/MASH is crucial [8,9,10]. Teodoro et al. analyzed rats fed with a choline-deficient diet (CDD) as an animal model of MAFLD and reported that mitochondrial respiratory function and the amount of adenine nucleotide translocator (ANT) were reduced following CDD feeding [8]. In addition, Xu et al. observed a decrease in the oxygen consumption rate in high-fat diet (HFD)-fed mice and changes in the expressions of genes, such as *CYP2E1*, in liver mitochondria [9]. Moreover, Romestaing et al. demonstrated decreased ATP levels and reduced ATP synthetic activity in the liver mitochondria of rats fed with a methionine/CDD (MCDD) [10]. However, the characteristics of the animal models used in these studies differ from those of MASH in humans in several ways. In HFD-fed mice, although fatty liver is observed, it does not progress to liver fibrosis. In CDD-fed mice, an extremely long time is required for liver fibrosis to develop, and distortions, such as cancer development in tissues other than the liver, occur with aging. MCDD-fed mice develop liver fibrosis that progresses to liver cancer, and they exhibit a marked decrease in body weight [11,12,13], which makes these animals less reflective of the pathogenesis of MASH in humans. To address this issue, a new MASH mouse model fed with a choline-deficient, methionine-defined, high-fat diet (CDAHFD) was recently reported [14]. Mice fed with CDAHFD develop liver fibrosis in a few weeks and HCC within 1 year without weight loss, indicating that this model closely resembles the pathogenesis of human MASH [14,15]

In this study, we analyzed the temporal functional changes in liver mitochondria in a MASH mouse model established via CDAHFD feeding and discussed the molecular mechanism of mitochondria-mediated MASH pathogenesis.

## 2. Results

### 2.1. Changes in Body Weight in CDAHFD-Fed Mice

**Figure 1:**
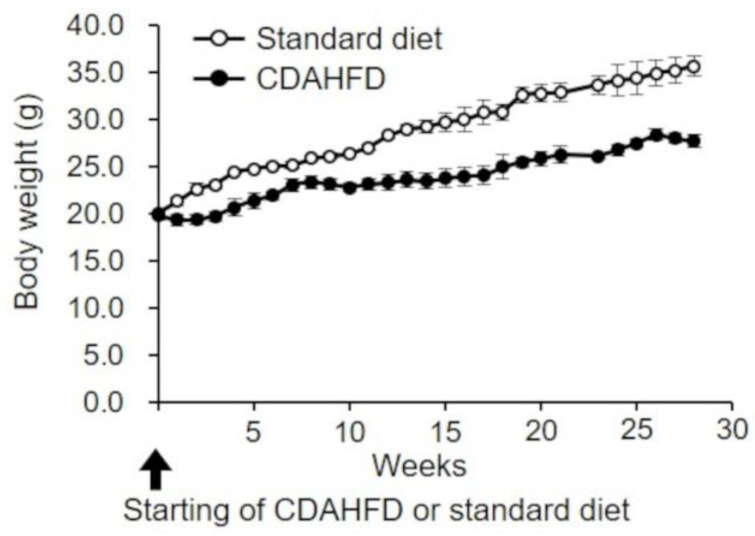
Body weight changes over time in CDAHFD-fed mice. Body weights of mice in the standard-diet (open circle) and CDAHFD (closed circle) groups were measured weekly. The mean ± standard deviation of the body weights in each group (n = 4 or 5) is shown. Standard diet, the mice group fed with standard diet; CDAHFD, the mice group fed with CDAHFD diet.

In this study, C57BL/6J mice fed with a standard diet were considered as the control, and those fed with CDAHFD served as the MASH model. The body weight of mice in both groups was approximately 20 g before the initiation of feeding. During the 28-week period from the start of feeding, the body weight of mice in the control group increased with aging, reaching approximately 36 g at 28 weeks. On the contrary, mice in the CDAHFD group showed a slight decrease in body weight 1–2 weeks after the start of CDAHFD feeding, followed by a moderate increase in body weight to approximately 28 g at 28 weeks (Figure 1).

### 2.2. Pathophysiological Analysis of the Livers of CDAHFD-Fed Mice

**Figure 2:**
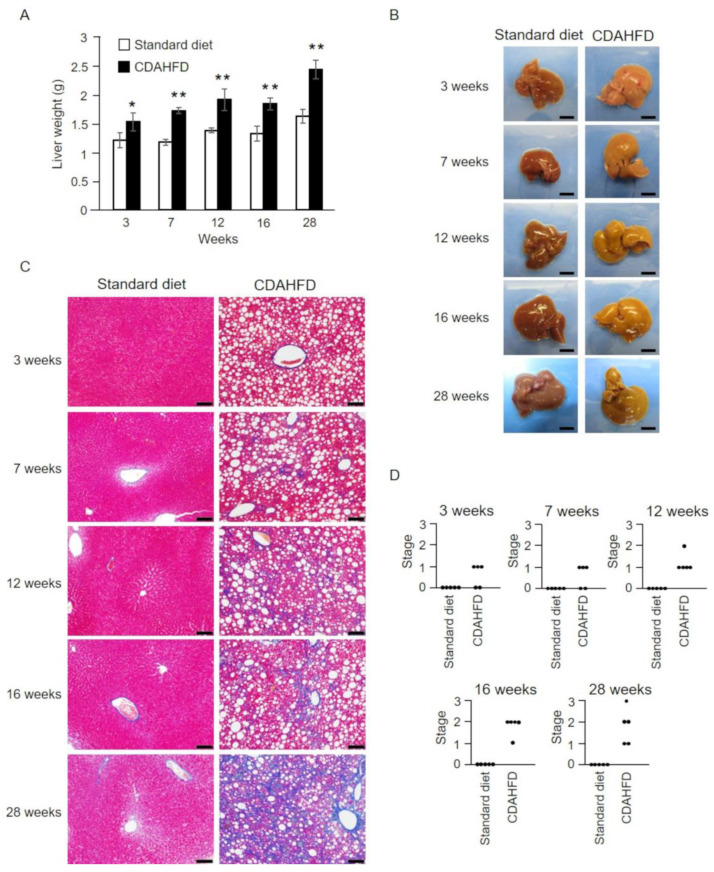
Pathological analysis of liver from CDAHFD-fed mice. (**A**) Livers were extracted from mice in the standard-diet (open square) and CDAHFD (closed square) groups and weighed over time. The mean ± standard deviation of liver weights for mice in each group (n = 4 or 5) was plotted. * *p* < 0.05, ** *p* < 0.01 (Student’s *t*-test). (**B**) Representative macroscopic images of the liver from mice in the standard-diet and CDAHFD groups at 3, 7, 12, 16, and 28 weeks (Scale bar = 10 mm). (**C**) Representative Masson’s trichrome staining of the liver from mice in the standard-diet and CDAHFD groups at 3, 7, 12, 16, and 28 weeks (Scale bar = 100 µm). (**D**) Liver fibrosis in mice in the standard-diet and CDAHFD groups: 0, normal liver sections without fibrosis; stage 1, fibrous expansion of the perisinusoidal or periportal area; stage 2, fibrous expansion of the perisinusoidal and periportal areas; and stage 3, bridging fibrosis. Standard diet, the mice group fed with standard diet; CDAHFD, the mice group fed with CDAHFD diet.

To analyze the liver pathology of CDAHFD-fed mice over time, livers were collected from mice at 3, 7, 12, 16, and 28 weeks of feeding and weighed. The results showed that liver weight was markedly increased after 3 weeks of CDAHFD feeding compared with that in the standard-diet group (Figure 2A). Subsequently, the liver weight significantly increased in the CDAHFD group at 7 weeks, and at 28 weeks it increased by approximately 1.5-fold of that in the standard-diet group (Figure 2A). In addition, the livers of mice in the CDAHFD group showed yellowish–white coloration after 3 weeks (Figure 2B), which is suggestive of fat accumulation, whereas those of mice in the standard-diet group remained brown throughout the analysis. Histopathological analysis confirmed the accumulation of liver fat in the CDAHFD group after a period as short as 3 weeks (Figure 2C and Figure S1). Moreover, the initial stages of liver fibrosis were evident in >50% of the mice at 3 weeks (Figure 2C,D). After 12 weeks, liver fibrosis was noted in all mice in the CDAHFD group, and the pathogenesis worsened with prolonged CDAHFD feeding (Figure 2C,D). Steatosis, inflammation, and hepatocellular ballooning, the major distinguishing features of MASH, were also observed (Figure S1). A mild degree of steatosis, inflammation, and ballooning were observed in the 28-week standard-diet group, but these were considered to be age-related changes.

### 2.3. Analysis of Oxidative Phosphorylation in the Liver Mitochondria of CDAHFD-Fed Mice

**Figure 3:**
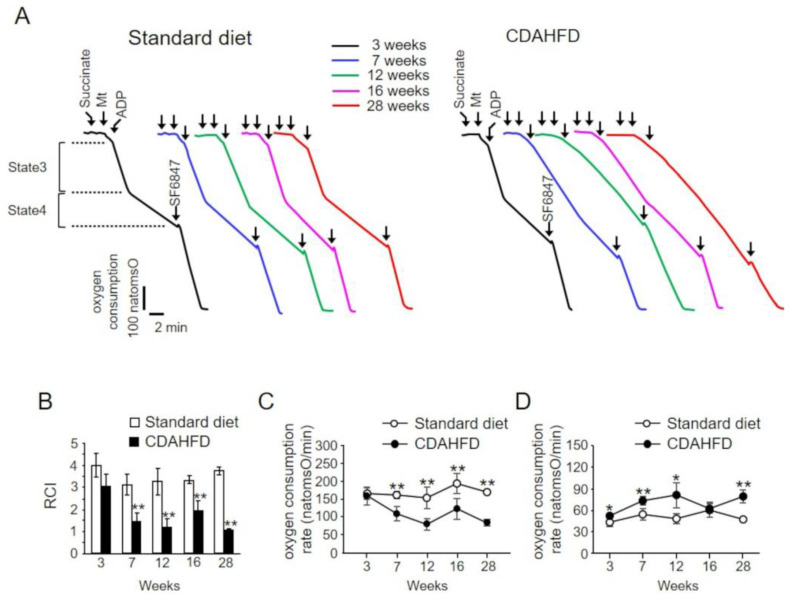
Functional evaluation of mitochondria isolated from the liver of CDAHFD-fed mice. (**A**) Oxygen consumption rate in mitochondria isolated from livers of mice in the standard-diet and CDAHFD groups. Mitochondria suspended in Pi medium were treated with 10 mM succinate, followed by the sequential addition of 250 µM ADP and 0.1 µM SF 6847. Results from the mitochondria isolated from the livers of mice in the standard-diet (**left panel**) and CDAHFD groups (**right panel**) are shown. Mitochondria isolated from mice in standard-diet and CDAHFD groups at 3, 7, 12, 16, and 28 weeks are shown in black, blue, green, pink, and red, respectively. The arrowheads indicate the points at which succinate, Mt, ADP, and SF6847 were added. Representative results from 3 to 5 mice per group are shown. Mt, mitochondria; natomsO, nano mol of oxygen atom in the reaction medium. (**B**) Respiratory control index (RCI) in the mitochondria isolated from the livers of mice in the standard-diet and CDAHFD groups. RCI was calculated as the state 3 oxygen consumption rate divided by the state 4 oxygen consumption rate shown in (**A**). The data represent mean ± standard deviations of 3–5 mice for each group. (**C**) State 3 in mitochondria isolated from the livers of mice in the standard-diet and CDAHFD groups. The data represent the mean ± standard deviations of 3–5 mice for each group. (**D**) State 4 in mitochondria isolated from the livers of mice in the standard-diet and CDAHFD groups. The data represent the mean ± standard deviations of 3–5 mice for each group. * *p* < 0.05, ** *p* < 0.01 (Student’s *t*-test). Standard diet, the mice group fed with standard diet; CDAHFD, the mice group fed with CDAHFD diet.

**Figure 4:**
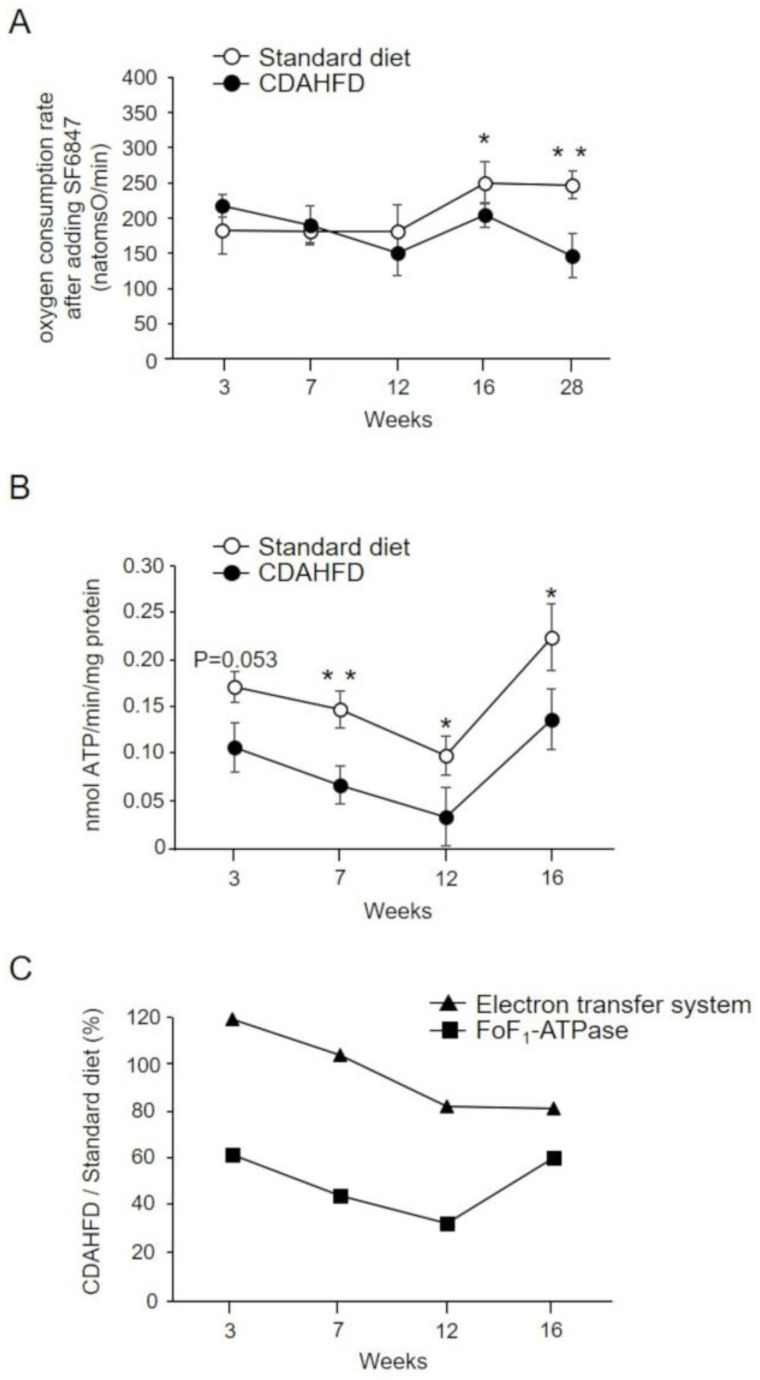
Evaluation of electron transfer system and FoF_1_–ATPase activity in mitochondria isolated from the livers of CDAHFD-fed mice. (**A**) Oxygen consumption rate in mitochondria isolated from the livers of mice in the standard-diet (open circle, n = 4–5) and CDAHFD (closed circle, n = 3–5) groups after the addition of SF6847. The data represent mean ± standard deviation. (**B**) The mitochondrial suspension was added to the incubation medium, followed by the sequential addition of 0.68 µg/mL rotenone, 10 mM succinate, and 250 µM ADP. The change in pH of the incubation medium was measured. The data represent the mean ± standard deviation of 3–5 mice for each group. (**C**) Electron transfer system (oxygen consumption rate after adding SF6847; triangle) and FoF_1_–ATPase activity (square) in the CDAHFD group relative to the standard-diet group (%). * *p* < 0.05, ** *p* < 0.01 (Student’s *t*-test). Standard diet, the mice group fed with standard diet; CDAHFD, the mice group fed with CDAHFD diet.

Oxygen consumption rate was measured in mitochondrial suspensions prepared from the livers of mice in the standard-diet and CDAHFD groups (Figure 3). For this analysis, succinate was used as the respiratory substrate, which supplied electrons to mitochondrial respiratory chain complex II. The addition of ADP resulted in a marked increase in the oxygen consumption rate coupled with ATP synthesis (state 3) and a subsequent slowing of the oxygen consumption rate associated with ADP depletion (state 4) in each feeding period in the control group (Figure 3A standard diet, traces after the addition of ADP). Furthermore, when the uncoupler SF6847 was added, an accelerated rate of oxygen consumption associated with the uncoupling of oxidative phosphorylation was observed in each feeding period (Figure 3A standard diet, traces after the addition of SF6847). By contrast, in liver mitochondria from mice in the CDAHFD group, an increase in the oxygen consumption rate was observed after the addition of ADP at 3 weeks. This finding was similar to those in mitochondria from mice in the standard-diet group (state 3). However, at 7 weeks the oxygen consumption rate slowed down with the addition of ADP. Although partial recovery was observed at 16 weeks, the rate decreased progressively with further extension of the feeding period (Figure 3A CDAHFD). In the CDAHFD group, the oxygen consumption rate after ADP depletion (state 4) was higher than that in the standard-diet group during all feeding periods, except at 16 weeks. Although the oxygen consumption rates following the addition of SF6847 decreased during the 16–28-week observation period in the CDAHFD group compared with those in the standard-diet group, the rates did not differ until 12 weeks between the standard-diet and CDAHFD groups (Figure 3A, traces after the addition of SF6847, and Figure 4A).

The respiratory control index (RCI) was calculated as the ratio of state 3 to state 4 measured in the traces. Mice in the standard-diet group displayed a high RCI, regardless of the feeding period, whereas mice in the CDAHFD group showed decreased RCI at 3 weeks compared with that in the standard-diet group. In addition, the RCI in the CDAHFD group was significantly lower at 7 weeks than that in the standard-diet group. Thereafter, although RCI recovered temporarily at 16 weeks, it decreased with further increase in the feeding period (Figure 3B). To determine the reasons for the decrease in RCI in the CDAHFD group, a quantitative evaluation was separately performed for state 3 and state 4. The results indicated that the oxygen consumption rate in state 3 did not change significantly between feeding periods in the standard-diet group (Figure 3C). On the contrary, oxygen consumption rate in state 3 at 3 weeks was similar in mice in the CDAHFD and standard-diet groups. However, after 7 weeks of feeding, mice in the CDAHFD group showed a decrease in their oxygen consumption rate in state 3. The oxygen consumption rate in state 3 in the CDAHFD group temporarily increased at 16 weeks, although it remained lower than that in the standard-diet group and decreased again at 28 weeks (Figure 3C). This temporary recovery at 16 weeks was in agreement with the results of a previous study [8]. Conversely, the oxygen consumption rate in state 4 was consistently higher in the CDAHFD group than in the standard-diet group from 3 weeks, except at 16 weeks (Figure 3D). A high level of state 4 implies increased proton permeability in the mitochondrial inner membrane. In addition, a temporary decrease in state 4 was observed at 16 weeks in the CDAHFD group; this decrease did not contradict the transient change in state 3 at 16 weeks. These findings indicate that the decrease in RCI in liver mitochondria in the CDAHFD group is caused by the additive effects of the decreased and increased oxygen consumption rates in state 3 and state 4, respectively. In addition, the early decrease in RCI at 3 weeks could be attributed to an increased oxygen consumption rate in state 4, suggesting that mitochondria from mice in the CDAHFD group exhibit an earlier increase in proton permeability in the inner membrane. To ascertain whether this early increase in state 4 at 3 weeks in the CDAHFD group is also induced under conditions in which electrons are supplied from mitochondrial respiratory chain complex I rather than complex II, the same experiment was performed with the addition of glutamic acid/maleic acid. The oxygen consumption rate in state 4 was increased in the CDAHFD group at 3 weeks compared with that in the standard-diet group, whereas there was no difference in the oxygen consumption rate in state 3 (Figure S2). This result is similar to that obtained after the addition of succinate as the respiratory substrate.

### 2.4. Analysis of FoF_1_–ATPase Activity in Liver Mitochondria Isolated from CDAHFD-Fed Mice

We measured FoF_1_–ATPase activity to determine if the cause of reduced oxygen consumption rate in state 3 was a dysfunction of the mitochondrial inner membrane electron transfer system or FoF_1_–ATPase. The oxygen consumption rate after the addition of SF6847 was comparable between the standard-diet and CDAHFD groups until 12 weeks, suggesting that the effect of the electron transfer system was negligible. After 12 weeks, the oxygen consumption rate after the addition of SF6847 in the CDAHFD group decreased compared with that in the standard-diet group with increasing feeding periods (Figure 4A). Conversely, the FoF_1_–ATPase activity in the CDAHFD group tended to be lower than that in the control group at 3 weeks, after which it remained consistently significantly lower than that in the standard-diet group, although there was a transient recovery at 16 weeks (Figure 4B). In the CDAHFD group, the activity of the electron transfer system remained approximately 80% of the activity of the standard-diet group, even at 16 weeks, while the FoF_1_–ATPase activity was already 62% of the activity of the standard-diet group at 3 weeks and then decreased in a feeding period-dependent manner to a significantly low 33% at 12 weeks (Figure 4C).

### 2.5. Analysis of the Calcium Retention Capacity (CRC) of Liver Mitochondria in CDAHFD-Fed Mice

**Figure 5:**
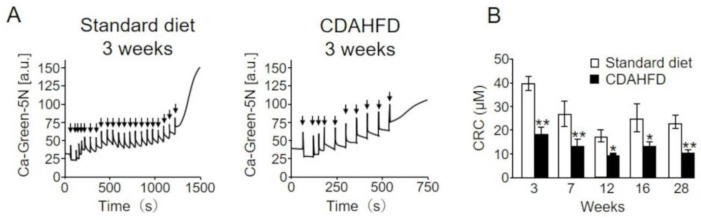
Calcium retention capacity (CRC) of liver mitochondria in CDAHFD-fed mice. (**A**) Isolated liver mitochondria of mice in the standard-diet (**left panel**) and CDAHFD (**right panel**) groups were incubated in an incubation medium with 1 µM calcium green (5 N), and 2 µM CaCl_2_ was gradually added until no further mitochondrial calcium uptake was observed at 3 weeks. Arrowheads indicate the addition of 2 μM CaCl_2_. (**B**) The total amount of Ca^2+^ added until Ca^2+^ was released from mitochondria into the incubation medium was measured. Open and closed squares represent the results of mice in the standard-diet and CDAHFD groups (n = 4–5 for each group). * *p* < 0.05, ** *p* < 0.01 (Student’s *t*-test). Standard diet, the mice group fed with standard diet; CDAHFD, the mice group fed with CDAHFD diet.

Mitochondria from mice in the CDAHFD group exhibited an increased oxygen consumption rate in state 4 and markedly reduced FoF_1_–ATPase activity compared with those in the standard-diet group (Figure 3 and Figure 4). These results indicate that the proton permeability of the inner mitochondrial membrane was increased in the CDAHFD group. Therefore, we evaluated the susceptibility of liver mitochondria from mice in the standard-diet and CDAHFD groups to the induction of nonsubstrate-selective permeability of the inner mitochondrial membrane (permeability transition (PT)). We repeatedly added low concentrations of calcium ions to the mitochondria isolated from the mice in both groups and examined the amount of calcium accumulated in the mitochondria until PT was induced (i.e., CRC), resulting in the release of calcium ions from the mitochondria (Figure 5A). The results illustrated that CRC was extremely lower in the liver mitochondria of mice in the CDAHFD group than in those of mice in the standard-diet group. The decrease in CRC occurred in a CDAHFD feeding-time-dependent manner. CRC tended to partially recover at 16 weeks, similar to the findings for FoF_1_–ATPase activity, RCI, and oxygen consumption rates (Figure 5B). Importantly, CRC was significantly decreased at 3 weeks after the initiation of CDAHFD feeding, indicating that liver mitochondria in CDAHFD-fed mice are susceptible to the induction of PT from an early stage.

### 2.6. Effects of Short-Term CDAHFD Feeding on the Absorbance of Mitochondrial Suspensions

**Figure 6:**
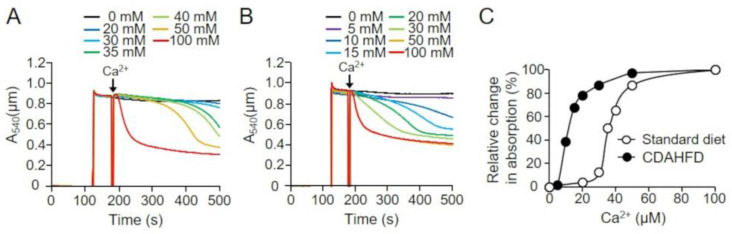
Ca^2+^-dependent absorbance changes in liver mitochondria of CDAHFD-fed mice. The absorbance was measured after the addition of various concentrations of Ca^2+^ to the isolated liver mitochondrial suspensions of mice in the standard-diet (**A**) and CDAHFD (**B**) groups at 3 weeks. (**C**) Relative changes to change in absorbance 320 s after the addition of 100 µM Ca^2+^ are shown. Open circle and closed circle are representative results of mice in the standard-diet and CDAHFD groups (n = 5 for each group), respectively. Standard diet, the mice group fed with standard diet; CDAHFD, the mice group fed with CDAHFD diet.

When PT is induced in mitochondria, the absorbance of the suspensions decreases because of mitochondrial swelling and inner membrane morphological changes [16,17,18]. Therefore, we analyzed the absorbance changes induced by Ca^2+^ addition in mitochondrial suspensions isolated from the livers of mice in the standard-diet and CDAHFD groups (Figure 6). When mitochondria from standard-diet-fed mice were analyzed at 3 weeks, a Ca^2+^ concentration-dependent decrease in the absorbance of the suspensions was observed (EC50 = 35.9 µM), confirming the Ca^2+^-dependent opening of permeability transition pore (PTP; Figure 6A,C, open circle). Furthermore, a decrease in absorbance was observed in mitochondrial suspensions obtained from the CDAHFD group, with an EC50 of 14.1 µM, which was significantly lower than that of the standard-diet group (Figure 6B,C, closed circle). These results were consistent with the outcomes of CRC analysis, indicating that PTP opening is more readily induced in mitochondria from mice in the CDAHFD group, even after 3 weeks of CDAHFD feeding.

### 2.7. Analysis of the Effects of Cyclosporin A (CsA) on the CRC of Mitochondria from CDAHFD-Fed Mice

**Figure 7:**
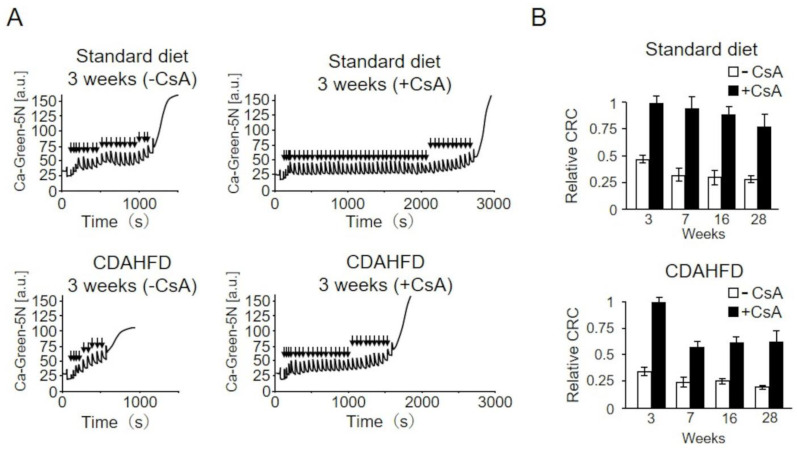
Effects of cyclosporin A (CsA) on the induction of permeability transition (PT) in liver mitochondria isolated from CDAHFD-fed mice. (**A**) CRCs were measured either without or with 1 µM CsA; upper traces indicate the results of mice in the standard-diet group and lower traces indicate those in the CDAHFD group at 3 weeks. Arrowheads indicate the addition of 2 μM CaCl_2_. (**B**) CRCs in isolated liver mitochondria of mice in the standard-diet and CDAHFD groups for various time periods of feeding were measured. The relative amount of CRC to the CRC with CsA at 3 weeks is shown. Data represent the mean ± standard deviation for each group (n = 4–5). Open and closed squares indicate the results without and with CsA, respectively. Standard diet, the mice group fed with standard diet; CDAHFD, the mice group fed with CDAHFD diet.

To further investigate the characteristics of PT induced in CDAHFD-fed mice, we examined the effects of CsA, a specific PT inhibitor (Figure 7). Liver mitochondria isolated from mice in the control group at 3 weeks required approximately 40 µM Ca^2+^ for PTP opening in the absence of CsA. Conversely, when CsA was added, approximately 90 µM Ca^2+^ was required for PTP opening, suggesting an elevated CRC and confirming that CsA inhibits PTP opening (Figure 7A upper trace). The suppression of PTP opening by CsA in the standard-diet group was observed consistently with extended feeding periods (Figure 7B). Conversely, in liver mitochondria from mice in the CDAHFD group, compared with no addition of CsA (white bars), the CRC considerably increased with the addition of CsA (black bars) at 3 weeks, as noted in the standard-diet group; however, the extent of increase reduced from week 7 (Figure 7A bottom trace, and B). This finding indicates that PTP opening induced in the liver mitochondria of mice in the CDAHFD group after 7 weeks was less sensitive to CsA, suggesting that CsA-insensitive PT occurs partially in MASH-induced liver mitochondria.

## 3. Discussion

In the present study, CDAHFD-fed mice were used as a MASH model to investigate the characteristics of liver mitochondria. The changes in body weight of CDAHFD-fed mice observed throughout this study were similar to those reported by Matsumoto et al. in a MASH mouse model developed using a similar CDAHFD feeding pattern [14], and this behavior mimicked the characteristics of patients with MASH [19] (Figure 1). Changes in liver weight and histopathology of CDAHFD-fed mice over time were also similar to those reported by Matsumoto et al. in CDAHFD-fed mice [14] and patients with MASH [20,21], confirming that the MASH mouse model was successfully established in this study via CDAHFD feeding (Figure 2). In this study, early signs of MASH (i.e., steatosis, inflammation, hepatocellular ballooning, and initial fibrosis) were observed in the livers of mice after 3 weeks of CDAHFD feeding (Figure 2 and Figure S1). In addition, the evaluation of several mitochondrial activities related to oxidative phosphorylation revealed that the activity of FoF_1_–ATPase decreased after 3 weeks of CDAHFD feeding (Figure 4B), although the behavior of respiratory activity in state 3 and the electron transfer system were not affected (Figure 3C, Figure 4A,C and Figure S2). These results suggest that mitochondrial dysfunction at an early stage of MASH related to oxidative phosphorylation is primarily attributable to the decrease in FoF_1_–ATPase activity. Moreover, an enhanced state 4 was observed in CDAHFD-fed mice as early as 3 weeks, regardless of whether the electrons were supplied from respiratory chain complex I or II. This indicates that the enhanced state 4 at an early stage of CDAHFD feeding is not caused by a respiratory chain complex-specific factor (Figure 3D and Figure S2B).

Our results showed that in CDAHFD-fed mice, PTP opening, which in turn reduces mitochondrial function, was accelerated early in the feeding period when FoF_1_–ATPase activity declined (Figure 5 and Figure 6). The increase in oxygen consumption rate in state 4 from 3 weeks may therefore be attributed to PTP opening (Figure 3D). These results suggest that FoF_1_–ATPase dysfunction and subsequent PTP opening are the initiating triggers for impaired mitochondrial function in the livers of CDAHFD-fed mice. Furthermore, mitochondrial dysfunction might be related to the induction and progression of liver fibrosis. The precise molecular mechanisms underlying PTP opening or regulation, which have not yet been clarified, will help understand the detailed molecular mechanisms of mitochondrial PT-mediated liver fibrosis progression in MASH.

Recently, Sugasawa et al. reported that CDAHFD feeding resulted in fatty liver pathology within 1 week, and at that time the expression levels of proteins related to mitochondrial energy metabolism, including those of FoF_1_–ATPase and mitochondrial respiratory complexes I and II, were reduced [22], although they did not perform functional analyses of these proteins. In contrast, in the present study, no significant effect on respiratory chain activity was observed after 3 weeks of CDAHFD feeding (Figure 3C, Figure 4A and Figure S2B). Further analysis is required to determine the effects of these reduced protein levels on mitochondrial function. Teodoro et al. performed a functional analysis of the liver mitochondria of MAFLD model rats fed CDD [8]. In the CDD group, during the first 4 weeks of CDD feeding, no decrease in FoF_1_–ATPase activity was observed compared with the control; however, at 8 weeks, FoF_1_–ATPase activity decreased by approximately 15%. Subsequently, at 16 weeks, no difference in FoF_1_–ATPase activity between control and CDD-fed rats was observed. Furthermore, the amount of ANT in the liver mitochondria of CDD-fed rats decreased during 12–16 weeks, and this decrease possibly contributes to the reduced mitochondrial oxidative phosphorylation capacity. In contrast, FoF_1_–ATPase activity was reduced by approximately 38% at 3 weeks after the start of CDAHFD feeding and by 55% at 7 weeks in this study (Figure 4B), suggesting that CDAHFD-fed mice showed a more pronounced reduction in FoF_1_–ATPase activity from an early stage than CDD-fed rats. In addition, Teodoro et al. demonstrated that mitochondrial function tended to be restored over time in CDD-fed rats [8] (the oxidative phosphorylation capacity was higher in rats fed CDD for 12 weeks than in rats fed CDD for 4 weeks). Although the degree of recovery was more gradual, the CDAHFD-fed mice in this study demonstrated a similar trend in the recovery of mitochondrial oxidative phosphorylation capacity at 16 weeks (Figure 3 and Figure 4). The reason for this transient recovery of mitochondrial function remains unclear; the elucidation of the whole landscape of changes in the liver mitochondrial proteome over time due to CDAHFD feeding may help us to understand the molecular mechanisms and physiological significance of the transient restoration of mitochondrial function.

To the best of our knowledge, this is the first report demonstrating that the reduction in FoF_1_–ATPase activity was accompanied by the promotion of mitochondrial PT in MASH models, as indicated by CRC (52% reduction at 3 weeks and 49% at 7 weeks in CDAHFD-fed mice (Figure 5B)). Several groups have investigated the relationship between FoF_1_–ATPase and PT [23,24,25,26,27,28,29]. Bernardi et al. reported PTP-like conductance in an FoF_1_–ATPase dimer, which was reconstituted by incorporating purified FoF_1_–ATPase into a lipid bilayer, and revealed that FoF_1_–ATPase is a major component of PTP [26]. In contrast, Walker et al. reported that the loss of FoF_1_–ATPase subunits in HAP1 cells had no effect on PTP opening [28]. Meanwhile, a recent study using HAP1 cells lacking the subunits g and F6 of FoF_1_–ATPase reported that these subunits were negative regulators of PTP opening [29]. Other reports have indicated that compounds that reduce the activity of FoF_1_–ATPase also play a role in the induction of PT [23,24,25], and regulators of endogenous FoF_1_–ATPase activity are involved in the regulation of PT [27]. For example, it has been reported that PT induction correlates with the expression level of mitochondrial ATPase inhibitory factor 1 (IF1), a regulator of FoF_1_–ATPase activity [27]. Thus, although the detailed mechanisms remain unclear, FoF_1_–ATPase is believed to be associated with the regulation of PTP opening. In this study, FoF_1_–ATPase activity (Figure 4) and CRC (Figure 5) were reduced early in the liver mitochondria of CDAHFD-fed mice, suggesting that some structural changes that alter the activity of FoF_1_–ATPase may have resulted in increased sensitivity to PTP opening, and the mitochondrial dysfunction caused by PT may contribute to the progression of MASH pathology. However, the cause for the early reduction in FoF_1_–ATPase activity in the pathogenesis of MASH remains unknown. Previously, Yang et al. used a mouse model of fatty liver fed on MCDD [12] and showed an increased production of reactive oxygen species (ROS) in the liver as the pathogenesis of MASH progressed; this suggests that ROS-induced FoF_1_–ATPase protein denaturation and oxidative modification may be involved in the decrease in FoF_1_–ATPase activity. However, further experiments are warranted to verify this hypothesis. Recently, it was shown that stimulation with palmitate increased the expression level of mitochondrial calcium uniporter (MCU) in AML12 mouse hepatocytes [30]. MCU is a protein that constitutes the channel domain of ion channels responsible for calcium ion intake into mitochondria and acts as an essential protein for mitochondrial calcium uptake together with EMRE [31,32,33,34]. Elevated expression levels of MCU may also induce increased calcium uptake into mitochondria, which may further promote PTP opening.

The findings of this study suggest that the use of PT inhibitors early in the pathogenesis of MASH may effectively control its progression. Currently, CsA [35], Debio-025 [36], and N-phenylbenzamides [37] have been identified as inhibitors of PT, and analysis of MASH pathogenesis in mouse models administered with these inhibitors can provide valuable insights. This study revealed that Ca^2+^-dependent PT induced in the liver mitochondria of CDAHFD-fed mice exhibited distinct characteristics compared with that in the liver mitochondria of standard-diet-fed mice. Thus, the present study demonstrated that CsA-insensitive PT is induced in a duration-dependent manner across the CDAHFD-feeding period. Previously, Yamamoto et al. reported that the application of cyanine dyes, such as DisC3-5 and TriS12; heavy metals, such as silver ion and cadmium; and the amphiphilic peptide mastoparan to the liver mitochondria of wild-type mice induced a partial CsA-sensitive PT at relatively low concentrations [17,38,39,40,41]. Although the mechanism underlying the induction of CsA-sensitive and -insensitive PT with different characteristics remains unclear, it is possible that mechanisms similar to the ones discussed above induce PT with different characteristics in the liver mitochondria of CDAHFD-fed mice. Further analysis is warranted to understand how CsA sensitivity changes with the duration of CDAHFD feeding. It is possible that ROS and other factors affect not only the structure and function of FoF_1_–ATPase but also the structure of cyclophilin D (CypD), the binding site of CsA, resulting in a change in the characteristics of PT, such as the sensitivity of CsA. The induction of CsA-insensitive PT was continuously observed from a relatively early stage of CDAHFD feeding (i.e., 7 weeks). Although there was no difference in the values of stage classification of liver fibrosis from 3 to 7 weeks (Figure 2D), histopathological analysis revealed that the area of fibrosis around the perisinusoidal or periportal region tended to increase at 7 weeks compared with that at 3 weeks, followed by progression of fibrosis (Figure 2C,D). Thus, the favorable correlation between the induction of CsA-insensitive PT and the progression of liver fibrosis suggests that this relatively prominent functional and structural change, which leads to insensitivity to CsA in mitochondria, plays a role in the pathogenesis of liver fibrosis in MASH. Therefore, the application of CsA and its derivatives may need attention in the development of drugs for MASH-targeting PTP. Compounds that can regulate PTP opening through targets other than CypD have been reported; hence, it is crucial to analyze the effects of these compounds on the pathogenesis of CDAHFD-fed mice. S15176 is an inhibitor of CsA-insensitive PT [39]; however, it cannot be used as a therapeutic agent by itself because it lowers the mitochondrial membrane potential at specific concentrations. The discovery of an analog of S15176 that reduces this effect and the analysis of the mechanism of PT inhibition by S15176 may enable the development of novel drugs for MASH that target the inhibition of CsA-insensitive PT.

In the present study, the following novel findings were revealed by analyzing liver mitochondria from CDAHFD-fed mice: (1) mitochondria showed decreased FoF_1_–ATPase activity preceding mitochondrial respiratory chain complexes, concurrent with the onset of liver fibrosis (i.e., 3 weeks after the initiation of CDAHFD feeding); (2) the decrease in FoF_1_–ATPase activity was followed by the promotion of PTP opening, for which FoF_1_–ATPase was one of the potential components or regulators—this led to the acceleration of mitochondrial dysfunction; and (3) as fibrosis progressed (i.e., after 7 weeks of CDAHFD feeding), the physical properties of PTP, i.e., CsA sensitivity, were altered. These findings suggest that the induction of fibrosis in MASH may be associated with mitochondrial dysfunction due to reduced mitochondrial FoF_1_–ATPase activity and PTP opening. The findings of this study might help in the elucidation of the mechanisms underlying the pathogenesis of MASH and the development of novel therapeutic strategies.

## 4. Materials and Methods

### 4.1. Animals

In total, 48 5-week-old male C57BL/6J mice (Japan SLC, Shizuoka, Japan) were acclimatized for 1 week under controlled light and humidity conditions with free access to food and water. Thereafter, the mice were divided into two groups: group fed with a standard diet (control group) and group fed with CDAHFD (MASH model group). The control diet (Certified Diet MF) was purchased from Oriental Yeast Co. (Tokyo, Japan), and CDAHFD (A06071302) was purchased from Research Diets Inc. (New Brunswick, NJ, USA).

### 4.2. Measurement of Body Weight

C57BL/6J mice were fed a standard diet or CDAHFD after acclimatizing them for 1 week. The weight of these mice was then measured on a weekly basis.

### 4.3. Anatomical and Histopathological Analyses of Liver Tissue

The livers were isolated from C57BL/6J mice fed either with a standard diet or CDAHFD. They were then weighed and observed. Masson’s trichrome staining was performed on paraffin-embedded liver samples to assess the level of hepatic fibrosis, and it was graded into stages 0–3 as follows: stage 0, normal liver sections without fibrosis; stage 1, fibrous expansion of the perisinusoidal or periportal area; stage 2, fibrous expansion of the perisinusoidal and periportal areas; and stage 3, bridging fibrosis. Hematoxylin and eosin staining was performed to analyze liver histology under a light microscope.

### 4.4. Isolation of Mouse Liver Mitochondria

Livers were isolated from C57BL/6J mice fed, either with a standard diet or CDAHFD, and mitochondria were isolated based on the method outlined in a previous report [42]. In brief, livers were finely minced with scissors and isolation solution (250 mM sucrose, 2 mM Tris-HCl, 1 mM EDTA-2Na, pH 7.40) was added. Then, the mixture was homogenized and centrifuged at 2500 rpm for 5 min. The resulting supernatant was further centrifuged at 7500 rpm for 10 min. After removing the supernatant, the precipitate was resuspended in the isolation solution. The suspension was then centrifuged at 12,000 rpm for 10 min, and the precipitate was resuspended twice in EDTA-2Na-free isolation solution, and the final suspension in EDTA-2Na-free isolation solution was used as the mitochondrial suspension. All centrifugation procedures were performed at 4 °C using a KUBOTA 6930 high-speed refrigerated centrifuge (KUBOTA, Tokyo, Japan). The mitochondrial protein concentration was determined using the bicinchoninic acid (BCA) method, with bovine serum albumin (BSA) as the standard in the presence of 1% sodium dodecyl sulfate.

### 4.5. Measurement of Mitochondrial Oxygen Consumption Rate

The mitochondrial oxygen consumption rate was measured using a Clark oxygen electrode (YSI5331, Yellow Springs Instrument Co., Yellow Springs, OH, USA). Pi medium (2.2 mL; (200 mM sucrose, 10 mM KPi, 2 mM MgCl_2_, 1 mM EDTA-2Na, pH 7.4)) was added to 0.42 mg protein/mL mitochondria, followed by the sequential addition of respiratory substrate, 250 µM ADP, and 0.1 µM SF6847, and the measurements were performed at 25 °C. When electrons were supplied by mitochondrial respiratory chain complex I, 5 mM glutamate and 5 mM malate were used as the respiratory substrates. When electrons were supplied by mitochondrial respiratory chain complex II, 0.68 µg/mL rotenone was previously added and 10 mM succinate was used as the respiratory substrate.

### 4.6. Measurement of FoF_1_–ATPase Activity

FoF_1_–ATPase activity was assessed by measuring pH changes in the incubation medium using a pH electrode, which is the classical method for measuring FoF_1_–ATPase activity [43]. The following reagents were sequentially added to the incubation medium (2.2 mL; (200 mM sucrose, 20 mM KCl, 3 mM MgCl_2_, 3 mM KPi, pH 7.40)): 0.68 µg/mL rotenone, 10 mM succinate, 0.42 mg protein/mL mitochondria, and 250 µM ADP. A pH electrode was used for measuring changes in the pH of mitochondrial suspension, and the assay was performed at 25 °C.

### 4.7. Mitochondrial Ca^2+^ Uptake Assay

Mitochondrial Ca^2+^ uptake was measured by adding 1 µM Calcium Green-5N, a calcium-sensitive fluorescent indicator, to 1 mL of incubation medium (0.3 M mannitol, 0.5 mg/mL BSA, 10 mM KPi, 2 mM NADH; pH 7.4 as respiratory substrate). Fluorescence intensity was measured at a wavelength of 532 nm using an F-2700 spectrofluorometer (HITACHI Co., Tokyo, Japan) with an excitation wavelength of 506 nm. CRC was measured by suspending mitochondria at a concentration of 0.2 mg protein/mL and adding 2 µM CaCl_2_ until no mitochondrial calcium uptake was observed. In the CsA condition, 1 µM CsA was previously added to the incubation medium.

### 4.8. Measuring the Absorbance of Mitochondrial Suspensions

Absorbance changes in mitochondrial suspensions were measured as previously reported and described [41]: 1 mL of Pi medium (200 mM sucrose, 10 mM KPi, 10 µM EGTA, pH 7.4) was sequentially added to 10 mM succinate, 0.35 mg/mL mitochondria, and 0–100 µM CaCl_2_, and the absorbance was measured at 540 nm using a spectrophotometer (UV-1800, Shimadzu, Kyoto, Japan).

### 4.9. Statistical Analysis

Data are expressed as mean ± standard deviation. The differences between two groups were assessed using an unpaired two-tailed Student’s *t*-test. The results were considered significant at *p* < 0.05.

## Data Availability

All data are contained within the article or Supplementary Materials.

## Supplementary Materials

The following supporting information can be downloaded at: https://www.mdpi.com/article/10.3390/ijms25116193/s1, Reference [44] is cited in the Supplementary Materials.
