# Supplementary material for: Longitudinal Analysis of Mitochondrial Function in a Choline-Deficient L-Amino Acid-Defined High-Fat Diet-Induced Metabolic Dysfunction-Associated Steatohepatitis Mouse Model
Source: Int J Mol Sci. 2024 Jun 4;25(11):6193. doi: 10.3390/ijms25116193 (PMC11173319; doi:10.3390/ijms25116193)
Supplement: Supplementary file 1 [file ijms-25-06193-s001.zip › ijms-2939656-supplementary.pdf]

Supplementary Materials

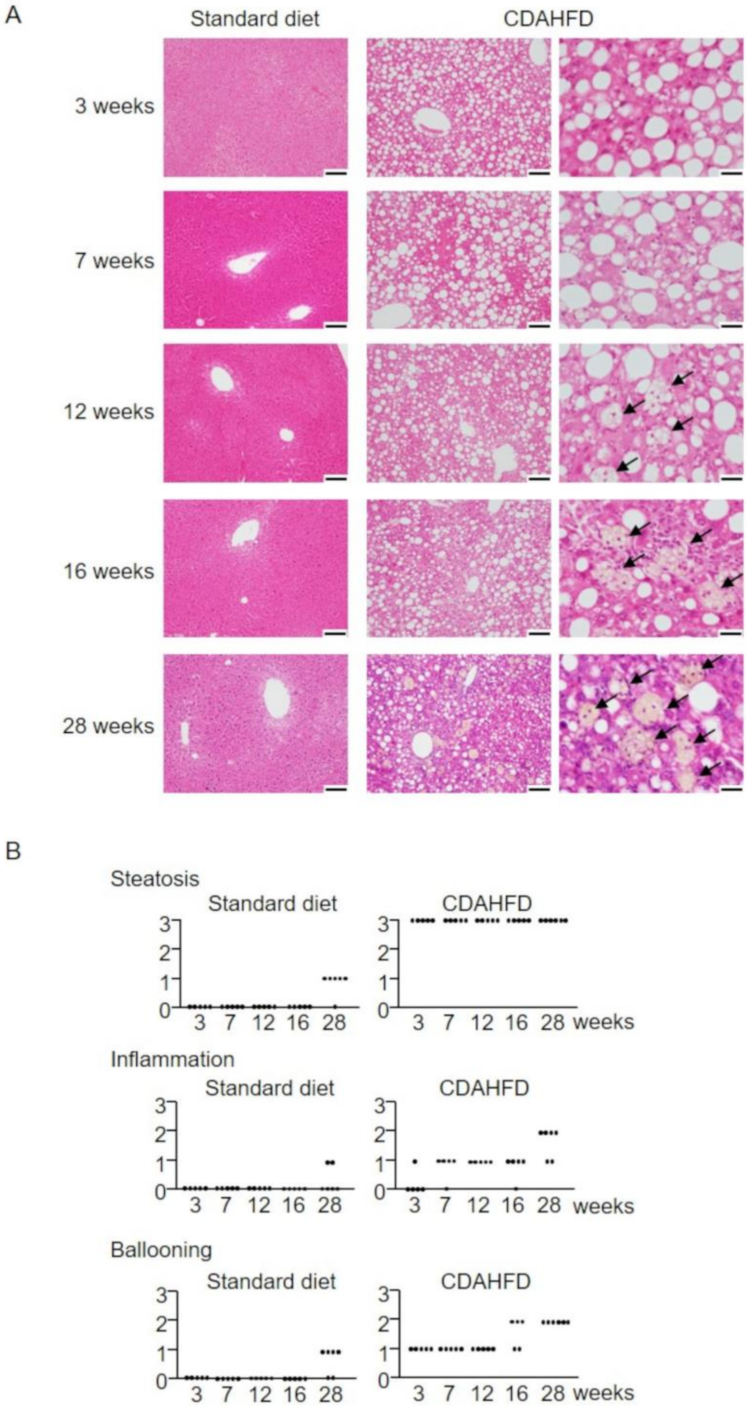

Figure S1. Histopathological evaluation of liver from CDAHFD-fed mice. (A) Hematoxylin and eosin staining of representative liver sections from mice in the standard diet (left panel) and CDAHFD (middle and right panels) groups at 3, 7, 12, 16, and 28 weeks. Left and middle panels; scale bar, 100 μm. Right panels; scale bar, 20 μm. Arrows indicate ballooned hepatocytes. Standard diet, the mice group fed with a standard diet; CDAHFD, the mice group fed with CDAHFD diet. (B) Hepatocellular steatosis, inflammation, and ballooning in mice in standard diet and CDAHFD groups: the morphological features of steatosis (stages 0–3), inflammation (stages 0–3), and ballooning (stages 0–2) were evaluated according to the activity score[44].

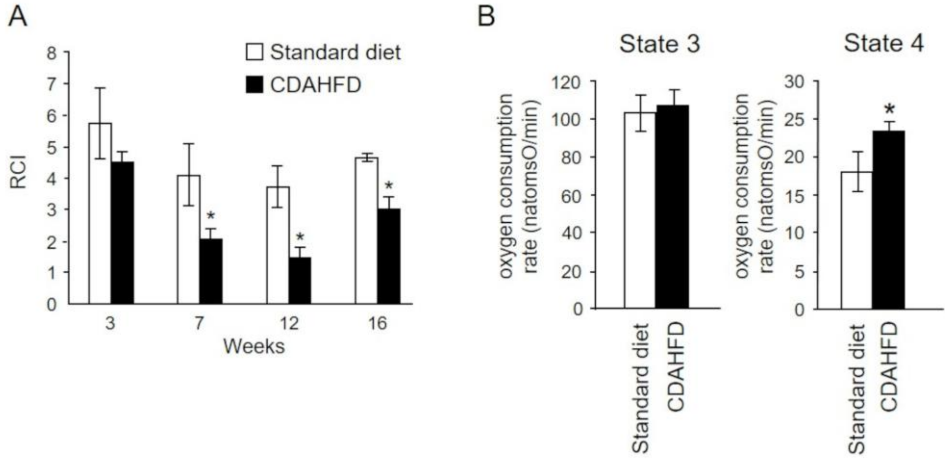

Figure S2. Evaluation of oxidative phosphorylation when electrons are supplied through mitochondrial respiratory complex I. (A) RCI in the mitochondria isolated from the livers of mice in the standard diet and CDAHFD groups. The data represent mean ± standard deviation of 3–5 mice for each group. (B) State 3 and 4 in the mitochondria isolated from the livers of mice in the standard diet and CDAHFD groups. The data represent mean ± standard deviation of 3–5 mice for each group. *$P < 0.05$ (student's t-test). Standard diet, the mice group fed with standard diet; CDAHFD, the mice group fed with CDAHFD diet.
